# Phylogenomics Reveals High Levels of Incomplete Lineage Sorting at the Ancestral Nodes of the Macaque Radiation

**DOI:** 10.1093/molbev/msad229

**Published:** 2023-10-12

**Authors:** Xinxin Tan, Jiwei Qi, Zhijin Liu, Pengfei Fan, Gaoming Liu, Liye Zhang, Ying Shen, Jing Li, Christian Roos, Xuming Zhou, Ming Li

**Affiliations:** CAS Key Laboratory of Animal Ecology and Conservation Biology, Institute of Zoology, Chinese Academy of Sciences, Beijing 100101, China; Geneplus-Beijing Institute, Beijing 102206, China; CAS Key Laboratory of Animal Ecology and Conservation Biology, Institute of Zoology, Chinese Academy of Sciences, Beijing 100101, China; College of Life Sciences, Capital Normal University, Beijing 100049, China; School of Life Sciences, Sun Yat-Sen University, Guangzhou 510275, China; CAS Key Laboratory of Animal Ecology and Conservation Biology, Institute of Zoology, Chinese Academy of Sciences, Beijing 100101, China; Primate Genetics Laboratory, German Primate Center, Leibniz Institute for Primate Research, Göttingen 37077, Germany; CAS Key Laboratory of Animal Ecology and Conservation Biology, Institute of Zoology, Chinese Academy of Sciences, Beijing 100101, China; Key Laboratory of Bio-resources and Eco-environment of Ministry of Education, College of Life Sciences, Sichuan University, Chengdu 610064, China; Primate Genetics Laboratory, German Primate Center, Leibniz Institute for Primate Research, Göttingen 37077, Germany; Gene Bank of Primates, German Primate Center, Leibniz Institute for Primate Research, Göttingen 37077, Germany; CAS Key Laboratory of Animal Ecology and Conservation Biology, Institute of Zoology, Chinese Academy of Sciences, Beijing 100101, China; CAS Key Laboratory of Animal Ecology and Conservation Biology, Institute of Zoology, Chinese Academy of Sciences, Beijing 100101, China

**Keywords:** *Macaca*, phylogenomics, incomplete lineage sorting, hybridization, radiation

## Abstract

The genus *Macaca* includes 23 species assigned into 4 to 7 groups. It exhibits the largest geographic range and represents the most successful example of adaptive radiation of nonhuman primates. However, intrageneric phylogenetic relationships among species remain controversial and have not been resolved so far. In this study, we conducted a phylogenomic analysis on 16 newly generated and 8 published macaque genomes. We found strong evidence supporting the division of this genus into 7 species groups. Incomplete lineage sorting (ILS) was the primary factor contributing to the discordance observed among gene trees; however, we also found evidence of hybridization events, specifically between the ancestral *arctoides/sinica* and *silenus/nigra* lineages that resulted in the hybrid formation of the *fascicularis/mulatta* group. Combined with fossil data, our phylogenomic data were used to establish a scenario for macaque radiation. These findings provide insights into ILS and potential ancient introgression events that were involved in the radiation of macaques, which will lead to a better understanding of the rapid speciation occurring in nonhuman primates.

## Introduction

The development of high-throughput sequencing technology and new computational tools for phylogenomics has enabled researchers to reconstruct the evolutionary histories of organisms by analyzing the distribution of inherited genetic markers in descendant lineages and looking back in time to identify those shared by a common ancestor (Figueiró et al. 2017; Árnason et al. 2018; Steenwyk et al. 2023). “Phylogenetic trees” generated from genomic data represent the most complete and best-supported hypotheses for the evolutionary history of a given organism; however, phylogenomic studies regularly reveal conflicting tree topologies, making it difficult to resolve certain branches in the tree of life (Whelan et al. 2017). Incomplete lineage sorting (ILS) is one potential explanation for gene tree discordances. ILS is primarily observed during rapid speciation events, when new lineages descend within a short time period from ancestors, particularly when there is a large effective population size (*Ne*) (Maddison and Knowles 2006; Avise and Robinson 2008). As a result, ILS causes ancestral polymorphisms to persist, even when descendant lineages have diverged (Szöllősi et al. 2015). Introgression and hybridization, however, are alternative explanations for gene tree discordances. Over the past decade, their role in evolutionary diversification has gained significant interest (Roos et al. 2011; Zinner et al. 2011; Abbott et al. 2013; Tung and Barreiro 2017; Fontsere et al. 2019; Van der Valk et al. 2020; Vanderpool et al. 2020). Hybridization is one of the most interesting topics in evolutionary biology. Genetic introgression caused by hybridization is not always maladaptive and may represent a potent evolutionary force (Abi-Rached et al. 2011; Abbott et al. 2013; Abbott et al. 2016; Zheng et al. 2020). Therefore, both ILS and gene flow or hybridization may result in topological incongruences between gene trees (the term “gene tree” is frequently used as shorthand for any locus in the genome, such as a protein-coding or protein-noncoding region) and species trees (Heled and Drummond 2010; Feng et al. 2022). For example, Edelman et al. (2019) used 20 de novo genome assemblies to explore speciation and gene flow in rapidly radiating *Heliconius* butterflies to distinguish ILS from introgression. They found that introgressed loci are underrepresented in low-recombination and gene-rich regions (Edelman et al. 2019). For primates, data for a genome-wide view on hybridization remain limited, but genome sequencing efforts are underway (Wu et al. 2022). For example, Rogers et al. (2019) and Sørensen et al. (2023) revealed complex reticulation in baboons and documented multiple episodes of admixture and introgression during the radiation of *Papio* using a comparative genomic approach.

**Fig. 1.**
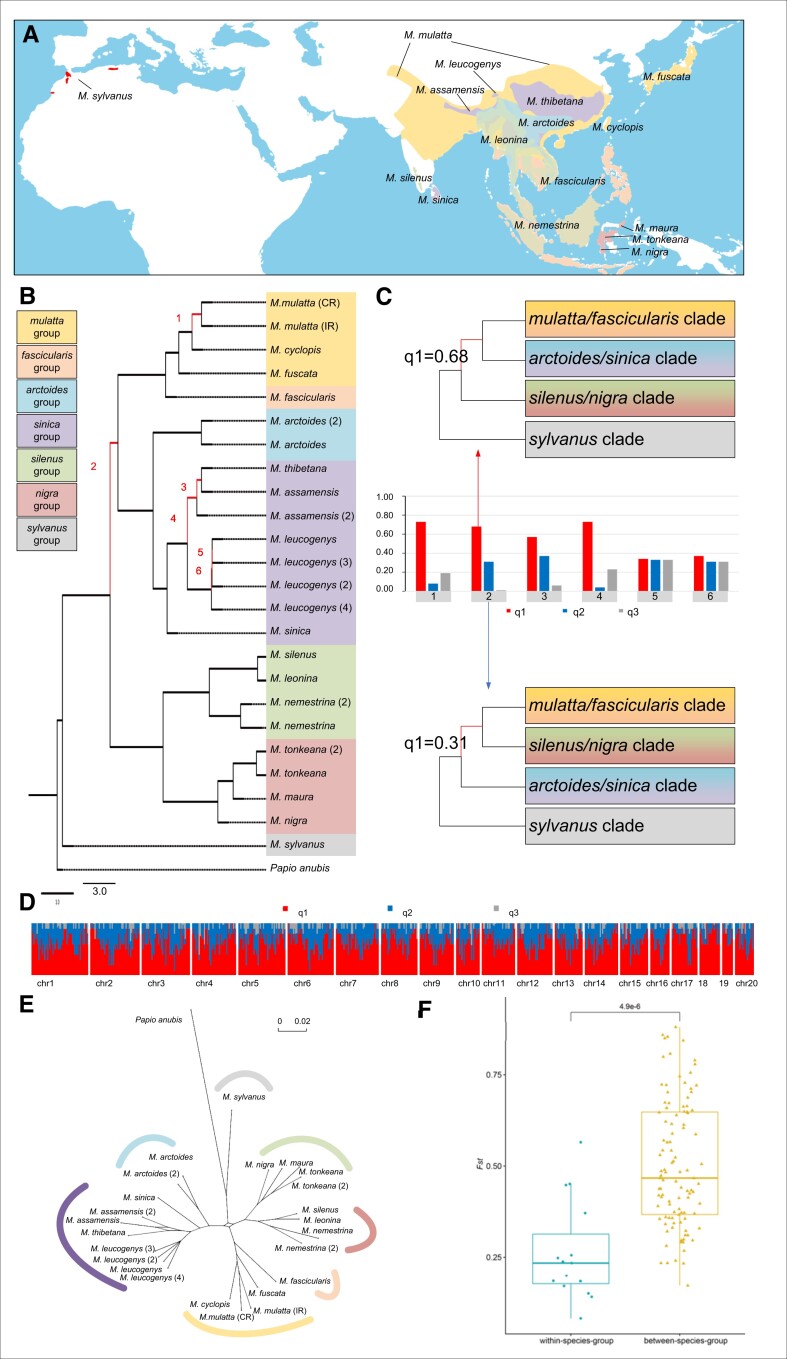
Macaque phylogenetics and differentiation. a) Distribution ranges of the 16 macaque species examined in this study. The range overlaps are shown in blended colors. b) An MSC species tree was constructed based on 6,351 individual GFs. Branches with a low q1 (<0.8) are numbered 1 to 6. All branches received maximal support (*P* = 1.0, ASTRAL analysis). Branch lengths were calculated from an ML analysis. Different species groups are represented by different background colors, which are show to the left. c) ASTRAL quartet-score analyses for branches 1 to 6 in b). Quartet scores were calculated for the 3 possible arrangements (q1 to q3) for the respective branches. The principal quartet trees are depicted, with q1 representing the species tree. Two alternative topologies for branch no. 2 as shown in c). d) Genealogical discordance across the genome of the rhesus macaque as demonstrated by a window analysis (200-kb window size) of a full-genome alignment mapped to Panu_3.0 (chromosomes shown at the bottom). The *y*-axis indicates the percentage of windows within a given interval (every 5 windows next to each other) that conform to (red) or reject (blue and gray) the species tree. e) Consensus network for macaques based on 6,351 GFs with a minimum threshold of 30% to form an edge. f) Distribution of *F*_ST_ within and between different macaque species groups (*P* = 0.05, Wilcoxon test).

The genus *Macaca* (Primates: Cercopithecidae) exhibits the largest geographic range and represents the most successful example of adaptive radiation of nonhuman primates (Zinner et al. 2013) (Fig. 1a). The 23 currently recognized macaque species may be subdivided into 4 to 7 groups according to differences in morphology, ecology, behavior, distribution, and genetics (Fooden 1976; Delson 1980; Morales and Melnick 1998; Groves 2001; Tosi, Disotell, et al. 2003; Sinha et al. 2005; Chakraborty et al. 2007; Zinner et al. 2013; Roos et al. 2014; Li et al. 2015; Hou et al. 2016; Fan et al. 2017; Roos et al. 2019) (Supplementary Fig. S1). However, the number and composition of these groups is controversial. Before the whole-genome area, phylogenetic analyses were primarily based on a few mitochondrial and/or nuclear markers, which resulted in contradicting phylogenetic relationships (Tosi et al. 2000; Tosi, Disotell, et al. 2003; Tosi, Morales, and Melnick 2003; Li, Handsaker, et al. 2009; Li, Han, et al. 2009; Fan et al. 2018). They were partially caused by introgression and hybridization events occurring among various macaque species.

Introgression and hybridization events between macaque species are common, for instance, among Sulawesi macaques, between *Macaca mulatta* and *Macaca fascicularis*, and between *M. mulatta* and *Macaca thibetana* (Ciani et al. 1989; Evans et al. 2001, 2003; Huson and Bryant 2006; Yan et al. 2011; Hamada et al. 2012; Fan et al. 2014; Evans et al. 2017; Ito et al. 2020). Moreover, for *Macaca arctoides*, a hybrid origin was suggested as different molecular markers placed the species into different clades (Tosi et al. 2000; Tosi, Morales, and Melnick 2003; Li, Handsaker, et al. 2009; Li, Han, et al. 2009; Fan et al. 2018; Roos et al. 2019). Most analyses, however, have focused on gene flow within macaque species groups or between species of the *fascicularis*/*mulatta* and *arctoides*/*sinica* species groups. Few studies include samples from all species groups for the identification of genus-wide hybridization and few attempted to identify ancient hybridization events in macaques (Fan et al. 2018; Matsudaira et al. 2018). A recent study described the history of the genus *Macaca* using 14 individuals from 9 species and detected extensive gene flow signals, with the strongest signals occurring between the *fascicularis/mulatta* and *silenus* groups (Song et al. 2021). Introgression signals among island species, such as Sulawesi macaques and other species, were also observed. This put forward a variety of possible reasons for this phenomenon including the genomic similarity of closely related species or ancestral introgression. Therefore, whether hybridization occurred among all macaques, especially among species that are geographically distant, still needs to be investigated. Furthermore, it remains unclear how much ancient hybridization or ILS has contributed to the diversity of the macaque genome.

In this study, we traced the complex, likely reticulated evolutionary history of macaques and newly sequenced whole genomes of 16 individuals representing 13 Asian species (*M. mulatta*, *Macaca cyclopis*, *Macaca fuscata*, *M. arctoides*, *Macaca assamensis*, *Macaca leucogenys*, *Macaca sinica*, *Macaca leonina*, *Macaca silenus*, *Macaca nemestrina*, *Macaca maura*, *Macaca tonkeana*, and *Macaca nigra*) and the only African macaque species (*Macaca sylvanus*). The sequences were mapped to the *Papio anubis* reference genome (Panu_3.0) to obtain single nucleotide variants (SNVs). Combined with 8 published macaque genomes, we performed a comprehensive genomics analysis of the 24 macaque individuals representing 16 species and all species groups. We reconstructed the phylogeny of the genus *Macaca*, investigated ILS and hybridization among the macaque species, and traced their evolutionary history and rapid radiation.

## Results

### Genome Sequencing and Variant Discovery

Whole-genome sequencing was performed using the Illumina HiSeq 2500 and 4000 platforms. Greater than 1,364 Gb of clean data (quality-controlled reads) were obtained, and the statistical results of the clean data for each species are listed in Supplementary Table S1. Published genome data for 8 macaques (*M. mulatta* [Chinese rhesus macaque, CR], *M. mulatta* [Indian rhesus macaque, IR], *M. fascicularis*, *M. arctoides*, *M. assamensis*, *M. thibetana*, *M. nemestrina*, and *M. tonkeana*) were downloaded from NCBI (Supplementary Table S1). Clean data for each species were mapped to the *P. anubis* reference genome (Panu_3.0) and yielded a genome-wide coverage of 16.85 to 42.34× with an average effective depth of 28.24× (Supplementary Table S1). We selected a more distantly related reference genome (*P. anubis*), rather than the rhesus macaque reference genome, to avoid any bias that may arise from mapping to an in-group reference genome. Although this may result in some data loss (in regions that lack orthologs in *P. anubis*), the mapping coverage (4×) for macaques ranges from 93.55% to 97.59%, which is very similar to the mapping coverage (4×) for *Papio* (98.75%), suggesting limited bias. The mapping rate for *M. nemestrina* (2) was less than 90%, which is likely the result of low DNA quality.

The number of high-quality SNVs for each individual ranged from 15,943,244 to 21,778,812, which included both homozygous and heterozygous alternatives (Supplementary Table S2). Genome-wide heterozygosity varied considerably among the macaques (Supplementary Fig. S2 and Table S2), which may partially reflect different demographic histories. Based on gene annotations from the reference genome, the exonic SNVs in macaques ranged from 225,336 to 257,712 (Supplementary Table S3). The species-specific SNVs for each macaque ranged from 1,279,649 to 8,688,344 (Supplementary Table S2).

### Phylogeny of Macaques

To reconstruct the phylogeny of macaques, we generated consensus sequences for genomes from all macaques. We split the aligned chromosomes into 6,351 nonoverlapping genome fragments (GFs) of 200 kb each, which represented 46% of the genome sequence. The multispecies coalescent (MSC) species tree based on the 6,351 GFs was supported with posterior probabilities (PPs) of 1.0 for all branches (Fig. 1b; Supplementary Fig. S3). This species tree, consistent with a neighbor-joining (NJ) tree and a maximum-likelihood (ML) tree based on autosomal SNVs (Supplementary Figs. S4 and S5), supported the segregation of macaques into 7 well-supported clades or lineages that corresponded to the classification of the genus into 7 species groups (Zinner et al. 2013; Roos et al. 2014). Among them, the African *sylvanus* lineage diverged first, followed by an initial separation of extant Asian macaques into a clade containing the *silenus* and *nigra* lineages and a clade including all other Asian lineages. The latter segregated into a clade including the *arctoides* and *sinica* lineages and another clade containing the *fascicularis* and *mulatta* lineages (Fig. 1b; Supplementary Figs. S3 to S5). The ML tree based on the mitochondrial genomes (mitogenomes), consistent with previous results (Fan et al. 2017; Roos et al. 2019), revealed that among the Asian macaques, the *silenus/nigra* clade separated first, followed by the *sinica* lineage. Of the remaining lineages, *fascicularis* split first, whereas *mulatta* and *arctoides* split afterwards (Supplementary Fig. S6 and Table S4). The quartet score “*q*,” which is a support value for possible phylogenetic arrangements, revealed a conflict in resolving the branch leading to the ancestor of the *fascicularis/mulatta* and *arctoides/sinica* groups (Fig. 1c and d, branch no. 2 in Fig. 1b; Supplementary Fig. S3).

A consensus network analysis of the GF trees revealed a network with bifurcating branches that emerged from a central cycle in the center of the network, indicating conflicting signals for the position of the *silenus/nigra* clade among Asian macaques (Fig. 1e; Supplementary Fig. S7). At a threshold of 30% for conflicting edges, the position of the *silenus/nigra*, *arctoides/sinica*, and *fascicularis/mulatta* clades could not be ascertained (Supplementary Fig. S7). When the threshold for conflicting edges was reduced, the phylogenetic signal was even more complex, indicating additional phylogenetic conflict (Supplementary Fig. S7). *F*_ST_ analyses suggested significant differences within (medians for *F*_ST_ = 0.199) and between (medians for *F*_ST_ = 0.467) the different macaque groups (*P* < 0.0001) (Supplementary Table S5 and Fig. S1f). A clear division of macaques into different clusters was also revealed by principal component analysis (PCA) (Supplementary Fig. S8 and Table S6).

### Contributions of ILS to Gene Tree Heterogeneity

**Fig. 2.**
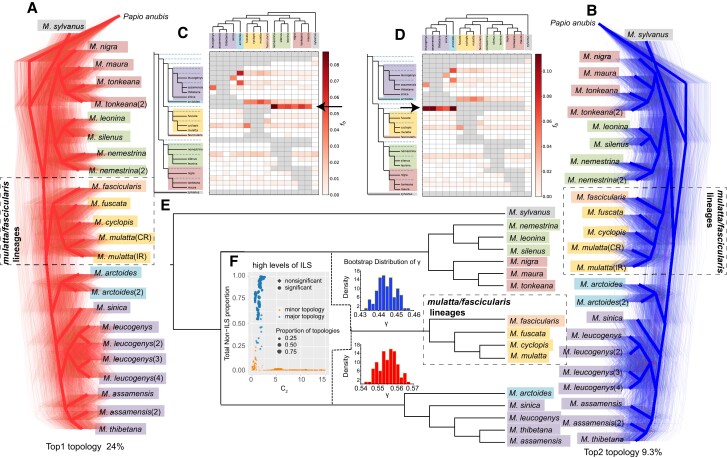
High levels of ILS and footprints of introgression contribute to gene tree heterogeneity. a, b) DensiTree plots based on 200-kb window trees spanning the whole genome. The top 2 most common topologies are represented by left a) and right b), respectively. The main incongruence between both trees is highlighted by the dashed boxes. c, d) The top 2 most common topologies were selected as reference trees and a heatmap is shown to illustrate the statistical support for introgression between species pairs inferred from Dsuit software. The *f*-branch function was used to further process the Dtrios results for every branch on the tree. Arrows indicate gene flow from either the ancestral *silenus/nigra* c) or *sinica/arctoides* d) clades into the ancestral *fascicularis/mulatta* clade. e) The diagram illustrates the complex evolutionary relationship between the macaque species, highlighting instances of hybridization and ILS. The *fascicularis/mulatta* clade is of hybrid origin, with the blue (*silenus/nigra*) and red (*sinica/arctoides*) *γ* corresponding to the estimated probabilities of inheritance from the respective ancestral clades. f) QuIBL results for 3 major Asian macaque clades. Relationships between the internal branch length in coalescent units (C_2_) and the total proportion of introgressed loci. Blue symbols represent triplets with the first most common topologies, and orange symbols represent triplets with the second most common topologies. The total proportion of introgressed loci was obtained by multiplying the probability that each topology corresponds to an introgression based on its genomic frequency. The figure shows that a large number of the most common topologies (i.e. the topology in a) have high non-ILS proportions, whereas the second most common topologies (i.e. the topology in b) has low non-ILS proportions when the internal branch lengths in coalescent units (C_2_) are short, which implies that the second most common topologies are primarily caused by ILS.

The topology cluster indicated that these 2 alternative topologies are the most common among all 6,351 GF trees (Figs. 1c and 2a and b). Our study aimed to investigate the extent to which the levels of gene tree heterogeneity observed in macaques may be attributed to ILS, particularly with respect to the phylogenetic conflict among the *fascicularis/mulatta*, *arctoides/sinica*, and *silenus/nigra* clades (Figs. 1c and 2a and b). To differentiate between ILS and introgression, we performed QuIBL, a recently developed tree-based method introduced by Edelman et al. (2019). QuIBL estimates the internal branch length distribution in discordant topologies for triplets of species and then calculates the likelihood that this distribution is consistent with either ILS and introgression or ILS alone. Using 3,175 trees (20-kb windows separated by 400-kb windows), we observed that among members of the *fascicularis/mulatta*, *silenus/nigra*, and *sinica/arctoides* clades, 73 of 120 tested triplets (60.8%) with internal branches exhibited phylogenetic discordances solely due to ILS (ΔBayesian information criterion [BIC] > 10). Furthermore, only 11 of the 120 triplets (9.1%) showed strong evidence of introgression (ΔBIC < −10) (Supplementary Table S7). For example, using QuIBL on the triplet *M. leonina*, *M. mulatta*, and *M. thibetana*, we inferred that only 1.33% of the loci across the entire genome were introgressed. Moreover, we found that a mere 0.29% of the genetic loci supported discordant topologies and were introgressed, indicating that interspecific introgression was limited among the species examined (Fig. 2f; Supplementary Table S7). Therefore, QuIBL analysis suggests that ILS, rather than introgression, is the predominant factor underlying the phylogenetic discordance among the species.

### Contribution of Hybridization to Gene Tree Heterogeneity

Previous studies have suggested a vital role of hybridization in shaping the evolutionary history of macaques (Ciani et al. 1989; Evans et al. 2001, 2003; Tosi, Disotell, et al. 2003; Hamada et al. 2006; Li, Handsaker, et al. 2009; Li, Han, et al. 2009; Yan et al. 2011; Hamada et al. 2012; Fan et al. 2014, 2017; Fan et al. 2018; Ito et al. 2020; Song et al. 2021; Zhang et al. 2023). In the present study, we inferred footprints of ancient introgression across macaques using an approach based on *D*-statistics (Durand et al. 2011) implemented in *Dsuite* (Malinsky et al. 2021) (Fig. 2a and b; Supplementary Table S8 to S9). This method estimates *D*-statistics for all possible combinations of trios in macaques based on the 2 alternative topologies shown in Fig. 2a and b and then performs an f-branch test to assign gene flow to specific internal branches (Fig. 2c and d). The *f*-branch test based on the species tree (Fig. 2a) suggested an introgression event between the ancestral *fascicularis/mulatta* and *silenus/nigra* lineages (Fig. 2c), whereas for the second most common topology (Fig. 2b), introgression between ancestral *fascicularis/mulatta* and *arctoides/sinica* lineages was evident (Fig. 2d). Next, we determined the number of reticulation events using the maximum pseudo-likelihood (MPL) algorithm in PhyloNet to study ancient hybrid speciation (Yu et al. 2014). This algorithm identifies reticulated nodes for the hybridization scenario between the ancestors of the *fascicularis*/*mulatta*, *arctoides*/*sinica*, and *silenus*/*nigra* lineages (Supplementary Fig. S9). Specifically, we observed an inheritance probability of approximately 30% between the *silenus*/*nigra* lineage and the *fascicularis*/*mulatta* lineage, which is consistent with the distribution of quartet scores and phylogenetic signals obtained from GF (log-likelihood scores = −10,786).

Next, we corroborated the evidence for the hybrid origin of the *fascicularis/mulatta* clade using hybridization detection (HyDe) (Blischak et al. 2018), which examines genome-scale data for a large number of taxa and identifies the population that may have arisen through hybrid speciation as well as its putative parental populations, by estimating an inheritance parameter (*γ* value)⁠ to quantify the genomic contribution of the parents to the hybrid. In the present study, we used HyDe to determine the significance level of hybridization for all clade combinations, including *P. anubis* as an outgroup and triplets of macaque in-group taxa. However, only 1 of the 4 significant hybridization events yielded a *γ* value of 0.55 (*Z*-score = 306.84; *P* = 0) (Supplementary Table S10), which indicates that the *fascicularis/mulatta* lineage is a hybrid product with genomic contributions of about 55% and 45% from the *arctoides/sinica* and *silenus/nigra* lineages, respectively (Supplementary Table S11). Next, we verified hybridization for the *fascicularis/mulatta* lineage at the individual level and found that all 5 individuals of this clade exhibited *γ* values of 0.534 to 0.571, further supporting the hybrid origin of the *fascicularis/mulatta* lineage (Supplementary Table S11). Finally, bootstrap resampling of individuals within hybrid populations was performed to obtain a distribution of gamma values to assess heterogeneity for the levels of introgression (Fig. 2e).

Finally, we used *F_d_* statistics (Martin et al. 2015) to calculate potential introgressed signals within 10-kb windows and considered the top 5% of the genomic regions with the highest *F_d_* values as potential introgressed regions. Given the challenge to accurately identify introgressed regions in macaque genomes, we applied a 2-layer hidden Markov model (Guan 2014) to infer the local ancestry of the admixed individuals of the *fascicularis/mulatta* lineage. We selected the top 1% of the windows with a high sum of the proportion of source population (*silenus/nigra* lineage) ancestry for all SNVs in 10-kb windows with high *F_d_* values as potential introgression regions (Supplementary Table S12) and identified some functional genes (e.g. *CERS3*, *ACER3*, and *GSK3B*) in these regions (Supplementary Fig. S10 and Table S13). Next, we reconstructed a phylogenetic tree based on autosomal SNVs including only the SNVs located in all putatively introgressed regions of the *fascicularis/mulatta* lineage that were derived from the *silenus/nigra* lineage. We observed that these lineages clustered together with the *arctoides/sinica* lineages to form their sister clade (Supplementary Fig. S11). We then reconstructed a phylogenetic tree excluding SNVs in these putatively introgressed regions and used only those in likely nonintrogressed regions to obtain a tree topology (Supplementary Fig. S11), which was identical to the species tree (Fig. 1b). Accordingly, trees based on introgressed and nonintrogressed regions resulted in different phylogenetic positions for the *fascicularis/mulatta* lineage.

### Divergence among Macaques

**Fig. 3.**
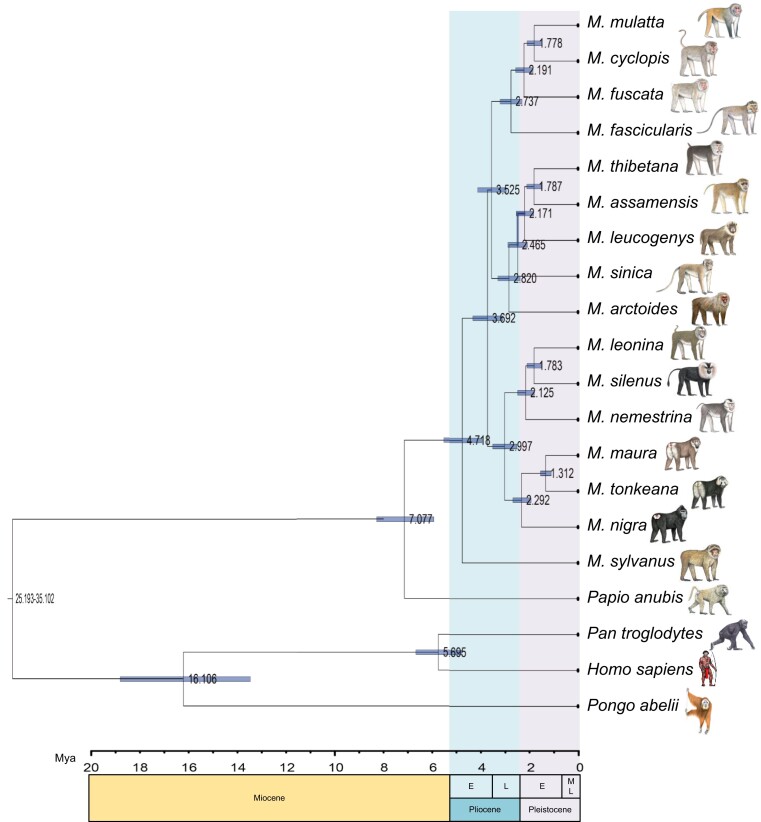
Phylogenetic tree showing divergence times among macaques that were estimated from 3,319,411 4D sites from 10,641 single-copy genes. Bars indicate 95% confidence intervals of divergence times and the time scale below indicates Ma. Four fossil-based calibration points are as follows: Catarrhini 20 to 38 Ma, Hominidae 13 to 18 Ma, *Homo*-*Pan* 6 to 7 Ma, and *Papio*-*Macaca* 5.33 to 12.51 Ma. E, early; L, late; M, middle.

Based on our divergence time estimations, the split between *Macaca* and *Papio* occurred in the Late Miocene period, approximately 7.08 Ma (95% highest posterior density [HPD] 8.21 to 5.88) (Fig. 3; Supplementary Fig. S12). Among the macaques, *M. sylvanus* diverged from Asian macaques at 4.72 Ma (95% HPD 5.47 to 3.92). In Asia, the divergence of species groups occurred in the Pliocene era, beginning with the split between the *silenus/nigra* clade and the other 4 species groups at 3.69 Ma (95% HPD 4.28 to 3.06), followed shortly afterward by the separation of the ancestors of the *fascicularis/mulatta* and *arctoides/sinica* clades (3.53 Ma, 95% HPD 4.1 to 2.93). These 3 clades finally diverged into species groups at 3 Ma (95% HPD 3.48 to 2.49; *silenus* and *nigra* groups), 2.82 Ma (95% HPD 3.27 to 2.34; *sinica* and *arctoides* groups), and 2.74 Ma (95% HPD 3.18 to 2.27; *mulatta* and *fascicularis* groups). Speciation within species groups occurred in the Pleistocene era at 2.46 to 1.31 Ma.

### Demographic History

Changes in the effective population size (*N_e_*) of the macaque species over time were modeled based on the distribution of heterozygous sites across the genome using a pairwise sequentially Markovian coalescent (PSMC) model (Li and Durbin 2011) (Supplementary Figs. S13a and d and S14). With the exception of *M. sylvanus* (Supplementary Fig. S13d), which exhibited a truncated plot most likely resulting from insufficient sequencing depth, the *N_e_* of the remaining macaque species was very similar from 5 Ma until ∼2.5 Ma. This indicates that all Asian macaque species shared the same demographic history during the Pliocene era. The Pliocene was a period of global cooling after the warmer Miocene era and the cooling and drying of the global environment may have contributed to the enormous spread of savannas during this time (Salzmann et al. 2011). The change in vegetation undoubtedly has a significant impact on arboreal animals, such as macaques. Subsequently, the ancestor of most macaque species exhibited an upward trend of *N_e_* after ∼2.5 Ma. However, the *N_e_* of 3 *nigra* group species remained stable until 1.5 Ma, with a subsequent upward trend at different rates, possibly corresponding to population growth and expansion once favorable ecological conditions were present. Macaques, except of the *nigra* group, experienced a population growth until the Pleistocene glaciations and the *Ne* of different macaque species declined during different glaciations, indicating that they were likely restricted to smaller refugia during glacial periods. During the last interglacial period, *N_e_* for *M. mulatta* (CR), *M. assamensis*, *M. nemestrina*, and *M. tonkeana* increased rapidly but subsequently declined again.

## Discussion

### Phylogeny and Classification of Species Groups

In the present study, we verified the validity of species groups within the *Macaca* genus and reconstructed their evolutionary history, which represents an exciting evolutionary scenario as a primate taxon, whose spread “Out of Africa” mirrors that of non-African humans. The species tree generated by ASTRAL supports the division of macaques into 7 well-supported clades or lineages that correspond to the classification of the genus into 7 species groups (Zinner et al. 2013; Roos et al. 2014) (Fig. 1b). *M. sylvanus* diverged first and the *F*_ST_ between *M. sylvanus* and Asian macaques was higher than among Asian macaque groups (Supplementary Table S5). These results support the separation of the *sylvanus* from the *silenus* group, thus confirming the classification of Delson (1980) based on morphology and geographic distribution. Asian macaques consist of *silenus* and *nigra* lineages and a clade including all other Asian lineages (Fig. 1b). The s*ilenus* and *nigra* lineages were grouped into a single species group (*silenus* group) by Fooden (1976) and Delson (1980) but were recently separated into 2 distinct species groups: *silenus* and *nigra* or Sulawesi (Groves 2001; Zinner et al. 2013; Roos et al. 2014, Roos et al. 2019). Based on the species tree, the 6 species of the *silenus* and *nigra* groups that we examined cluster into 2 clades: *silenus* group (*M. silenus*, *M. leonina*, and *M. nemestrina*) and *nigra* group (*M. nigra*, *M. maura*, and *M. tonkeana*) (Fig. 1b).

The other Asian macaques may be divided into 2 major clades. The first one includes *M. fascicularis*, *M. fuscata*, *M. cyclopis*, and *M. mulatta* (Fig. 1b). Initially, these 4 species were grouped into the *fascicularis* group (Fooden 1976); however, Groves (2001) separated *M. fuscata*, *M. cyclopis*, and *M. mulatta* from the *fascicularis* group into the own species group, *mulatta* group. The *F*_ST_ values varied widely between *M. fascicularis* versus *M. mulatta* (0.171) and *M. fascicularis* versus *M. fuscata* (0.415). Yan et al. (2011) indicated that approximately 30% of the genomes of mainland *M. fascicularis* are of Chinese *M. mulatta* origin, which explains the lower genetic distance between these 2 species in our analysis. However, *M. fascicularis* from the Sundaland region may not contain any genomic contribution of *M. mulatta*, which likely results in increased *F*_ST_ values, thus justifying to separate them into distinct species groups. The second major clade consists of *M. arctoides*, *M. assamensis*, *M. thibetana*, *M. leucogenys*, and *M. sinica* (Fig. 1b). *M. arctoides* was recently assigned to its own species group, the *arctoides* group (Zinner et al. 2013; Roos et al. 2014, 2019), but due to morphological similarities traditionally classified as a member of the *sinica* group (Delson 1980; Tosi et al. 2000; Li, Handsaker, et al. 2009; Li, Han, et al. 2009), while Groves (2001) recognized *M. arctoides* as a member of the *fascicularis* group. *M. arctoides* should be separated from the *sinica* group in its own species group.

### Contributions of ILS and Ancient Hybridization to Gene Tree Heterogeneity

In a recent study, gene flow between the *fascicularis/mulatta* and *silenus/nigra* groups was reported. It was concluded that the introgression signals between both groups are primarily the result of genome similarity in closely related species (Song et al. 2021). In the present study, we used QuIBL to show that among 120 triplets, 60.8% exhibited phylogenetic discordances exclusively resulting from ILS. Moreover, evidence for introgression was only observed in 9.1% of the triplets (Supplementary Table S7), indicating that ILS, rather than introgression, is the primary factor that contributes to the observed gene tree discordance. However, our results also suggest that ancient hybrid speciation may have occurred among the ancestral populations of the *silenus/nigra* and *arctoides/sinica* clades. This resulted in the formation of the *fascicularis/mulatta* clade, although these analyses do not differentiate between ILS and introgression, which can both create similar phylogenetic signals (Fontaine et al. 2015). Thus, a plausible explanation is that the *fascicularis/mulatta* clade may have originated from an ancient hybrid speciation of 2 parental lineages (the ancestral populations *silenus/nigra* and *arctoides/sinica* clades), which was undoubtedly accompanied by a high level of ILS. The reason why we are confident in the notable incidence of ILS is that, according to PSMC analysis, all species in the genus *Macaca* experienced a population decline during the time window of the potential hybridization event (3.53 to 3.69 Ma). Population declines in ancestral lineages result in rapid segregation into small isolated subgroups resulting in a rapidly diverging lineage. Furthermore, the substantial fraction of gene trees that are incongruent with the species tree in the genome, also referred to as hemiplasy (Avise and Robinson 2008), is particularly prevalent when ancestral populations are large and the time interval between speciation events is short. Hemiplasy may be attributed to the stochastic segregation of ancestral allelic polymorphisms from ancestrally large effective populations into already diverging subpopulations within a relatively short time interval (Whitfield and Lockhart 2007; Steenwyk et al. 2023). Thus, all of this constitutes a prerequisite for high levels of ILS at the ancestral nodes of the Asian macaque radiation. With respect to potential hybridization events, our *Dsuit* results based on the 2 most common tree topologies (Fig. 2c and d), PhyloNet (Supplementary Fig. S10) and HyDe (Fig. 2e; Supplementary Tables S10 and S11), suggest the *fascicularis/mulatta* clade as a potential hybrid product. However, the authenticity of this event remains under discussion because of 2 reasons. First, it is difficult to accurately detect the superposition of multiple introgression events throughout the evolutionary history of macaques, and ancient hybridization events may have subsequent unique evolutionary histories, such as ongoing gene flow, distinct introgression histories, maintenance of assortative mating, and rate heterogeneity (Sankararaman et al. 2014). Second, the power of HyDe and *D*-statistics to detect hybridization events in the presence of high rates of ILS and ancient rapid radiation events is limited (Kong and Kubatko 2021). Simulation analyses revealed that in the case of high levels of ILS, the power of HyDe decreases to 0 to 0.1, and the power of *D*-statistics was relatively poor (0.03 to 0.7) either. Furthermore, the accuracy of HyDe for the *γ* estimates of hybridization events relative to ancestry is further reduced and accompanied by a high false-positive rate (Kong and Kubatko 2021). The reason therefore is that ILS contributes to both ABBA and BABA counts, thereby diluting information and masking the signature of hybridization in the hybrid genome. Individuals or taxa with longer external branch lengths have more time to accumulate mutations, and the specific pattern of loci that are informative for detecting hybridization may be lost (Kong and Kubatko 2021). Although the authenticity of hybridization cannot be confirmed in the present study, we believe that both the high levels of ILS and potential hybridization contributed to the genealogical discordance observed across the genome. Our study and that of others show that macaque individuals, even from the same species and population, can exhibit quite distinct genetic backgrounds (Xue et al. 2016; Liu et al. 2018). For a better understanding of macaque evolution and the extent of hybridization, more individuals should be evaluated in future studies.

### The Radiation of Macaques

Fossil data suggest that the genus *Macaca* arose about 7 Ma in Northern Africa during the Late Miocene era (Delson 1980; Whitfield and Lockhart 2007; Salzmann et al. 2011; Fontaine et al. 2015), which is consistent with our MCMCTree results (7.08 Ma; Fig. 3). The Messinian salinity crisis (5.9 to 5.3 Ma), in which the level of the Mediterranean Sea fluctuated and occasionally dried out, may have facilitated macaque dispersal from North Africa (Hamada et al. 2006; Elton and O'Regan 2014). A large number of macaque fossils have been discovered in Europe near the Mediterranean (Hamada et al. 2006; Roos et al. 2019). Macaque ancestors likely invaded Europe via the arid Mediterranean during Messinian stage (Tosi, Disotell, et al. 2003; Tosi, Morales, and Melnick 2003; Hamada et al. 2006; Roos et al. 2019). The *M. sylvanus* ancestor split from the main stem at ∼4.72 Ma and settled around the Mediterranean (Fig. 3; Supplementary Fig. S15). Dental specimens from the oldest known Asian fossil, referred to as the genus *Macaca*, are *Macaca palaeindicus* from the Late Pliocene Tatrot formation of the Siwalik Hills (Delson 1980; Salzmann et al. 2011; Sankararaman et al. 2014). Hence, the ancestor of Asian macaques spread eastward step by step likely along the Siwalik Hills to the Hengduan Mountains, which are a potential diversification hotspot for primates (Peng et al. 1993; Jablonski 1998; Thinh et al. 2010; Roos et al. 2011), and diverged into different lineages. First, the ancestor of *silenus/nigra* split at ∼3.69 Ma and then potentially hybridized with the ancestor of other Asian macaques and formed a pattern of 3 clades (Supplementary Fig. S15).

## Conclusion

We conducted a phylogenetic analysis of 24 whole macaque genomes, of which 16 were newly generated. We identified ∼20 million high-quality SNVs for each macaque individual, which can be used as important baseline data for future studies. We found support for the division of macaques into 7 species groups and observed visible differences in *F*_ST_ within and between species groups. We also conclude that ILS is the main factor that caused gene tree discordances in macaques, rather than introgression. Specifically, the conflicting phylogenetic position of the *fascicularis*/*mulatta* is likely the result of high levels of ILS and potential hybridization between ancestors of the *arctoides*/*sinica* and *silenus*/*nigra* lineages. In addition, we used the available fossil data along with the literature to reconstruct the radiation scenario of the macaques.

## Materials and Methods

### Sample Collection and Library Preparation

Samples of 16 macaque individuals were obtained from the Institute of Zoology, Chinese Academy of Sciences and the German Primate Center (Supplementary Table S1). Genome data for 8 other macaque individuals and 1 *P. anubis* individual were obtained from the NCBI Short Read Archive (for accession number, see Supplementary Table S1). The present study was approved by the Animal Ethics Committee of the Institute of Zoology, Chinese Academy of Sciences. The procedure for blood and tissue collection was in strict accordance with the Animal Ethics Procedures and Guidelines of the People's Republic of China. DNA was isolated from the blood or tissue samples using the Qiagen Blood and Tissue Kit. Sequencing libraries were prepared based on the KAPA library preparation kit with insert sizes of 300 to 500 bp and sequenced using Illumina HiSeq 2500 and 4000 technologies. Quality control was performed using FastQC with default parameters (www.bioinformatics.babraham.ac.uk/projects/fastqc/).

### Data Processing

Paired-end reads for all samples were mapped to the *P. anubis* reference genome (Panu_3.0, GCA_000264685.2) using the Burrows–Wheeler Aligner mem v0.7.17 (Li and Durbin 2009), except for *M. nemestrina* (2), which was trimmed with cutadapt v2.4 (Kechin et al. 2017) first, because of poor sequencing quality resulting from DNA degradation. The output bam files were sorted using SAMtools (Li, Handsaker, et al. 2009; Li, Han, et al. 2009), and duplicates were marked with GATK v4.1.2.0 (McKenna et al. 2010). SNVs were called using GATK following best practice. We obtained a GVCF file for each individual using the “HaplotypeCaller” method in GATK, and the samples were merged based on “CombineGVCFs.” We used the GenotypeGVCFs-based method with the “includeNonVariantSites” flag to obtain the vcf files. Next, we applied “SelectVariants” to exclude indels and split the variant and nonvariant sites. All nonvariant sites were filtered in subsequent analyses. We applied the hard filter command “VariantFiltration” to exclude potential false-positive variant calls using the following criteria: “-filterExpression QD < 5.0 || FS > 60.0 || MQ < 40.0 || ReadPosRankSum < −8.0 || MQRankSum < −12.5” and “-genotypeFilterExpression DP < 4.0” (Van der Auwera et al. 2013). All variant calls failing the filter were removed. All SNVs were annotated using ANNOVAR v2013–06-21 (Wang et al. 2010) (Supplementary Table S3) based on the gff3 file from Ensembl (Papio_anubis.Panu_3.0.104.gff3). Heterozygosity was defined as the rate of heterozygous SNVs in the genome, which were further filtered using the following parameters: “--minGQ 20” and “--minDP 8” to ensure data quality based on VCFtools. We calculated the heterozygosity rate in nonoverlapping windows of 100 kb in size for all 24 macaques. The genome-wide distribution of heterozygosity is shown in a box plot using boxplot from the R package (Supplementary Table S2 and Fig. S2).

### Phylogenetic Reconstruction Using a Window-Based Strategy

We constructed consensus sequences for all individual genomes and aligned them per chromosome (sex chromosomes, mitogenomes, and scaffolds were excluded) based on VCF, which did not include variants present in the *Papio* reference, but were invariant among macaques. In addition, indels, repetitive regions, and low-quality regions were removed from the consensus sequences. Per-chromosome alignments were split into nonoverlapping windows of 200 kb. For each window, we constructed ML trees with RAxML v8 (Stamatakis 2014) using the GTR + CAT substitution model and 100 bootstrap replicates. Using the 200-kb window trees, we reconstructed a species tree using ASTRAL v4.10.5 (Sayyari and Mirarab 2016) under the MSC model returning quartet scores and PPs. The species tree was rooted with *P. anubis*. Consensus networks of the window trees were generated using SplitsTree5 (Huson and Bryant 2006) using different thresholds. Genealogical discordance across the rhesus macaque genome was assessed by Twisst (Martin and Van Belleghem 2017). All GF trees were clustered using PhyBin (https://github.com/rrnewton/PhyBin), and the top 2 topologies were visualized by DensiTree (Bouckaert 2010).

### Phylogenetic Analysis of Concatenated Whole-Genome SNV Data

SNVs were extracted from the filtered VCF file and converted into individual FASTA sequences using a python script (https://doi.org/10.5281/zenodo.2540861). The FASTA sequences were merged into a single file to provide a multiple sequence alignment for all individuals and all concatenated SNVs. An ML phylogenetic tree was reconstructed with RAxML v8 using the GTR + CAT substitution model and 100 bootstrap replicates. In addition, a NJ tree was constructed using the TreeBeST v1.9.2 program (http://treesoft.sourceforge.net/treebest.shtml), which has a built-in algorithm to generate the best tree that reconciles with the species tree and is rooted with the minimal number of duplications and losses. To further visualize genetic relationships among the macaques, we performed PCA on the filtered autosomal SNVs using the Eigensoft package v5.0 (Patterson et al. 2006).

### Phylogeny of Mitochondrial Genomes

We used NOVOPlasty (Dierckxsens et al. 2017) to de novo assemble the mitochondrial genomes (mitogenomes) of 18 macaques representing 15 species (Supplementary Table S4) and annotated them with MitoZ (Meng et al. 2019). In addition, we downloaded 15 additional macaque mitogenomes (Supplementary Table S4) from the NCBI based on the filter criteria mentioned in Roos et al. (2019). The 33 mitogenomes were aligned using MAFFT v7 (Yamada et al. 2016) and indels and poorly aligned positions were removed with Gblocks v0.91b (Castresana 2000). An ML tree was reconstructed using RAxML v8 with a GTR + CAT substitution model and 1,000 bootstrap replicates.

### QuIBL

QuIBL (Edelman et al. 2019) was used to determine the likelihood of introgression and ILS for each locus in all species triplets. QuIBL provides estimates of the proportion of introgression and the probability of a locus falling into a model with either only ILS or introgression and ILS. QuIBL estimates the internal branch length distribution for each locus in the species triplet. Given its sensitivity to recombination (Edelman et al. 2019), we extracted 20-kb windows separated by 400 kb to minimize the risk of including a recombination breakpoint in the window. We then filtered the inferred ML trees based on the number of parsimony-informative sites (≧10), which resulted in a total of 3,175 trees that served as QuIBL inputs. We used the species tree topology to assign an outgroup to each triplet and calculated the percentage of loci that supported discordant topologies and showed significant evidence of introgression.

To determine whether ILS (scenario 1) or a combination of ILS and introgression (scenario 2) contributed to the observed gene tree discordances, we calculated QuIBL delta BIC values for inner branch lengths. To determine the most suitable model, differences in delta BIC were calculated by subtracting the delta BIC value of scenario 1 from the delta BIC value of scenario 2. When the difference in the delta BIC score is greater than 10, scenario 1 (only ILS) is preferred. If less than −10, scenario 2 (ILS and introgression) is preferred. When the difference is in the range of −10 to 10, both scenarios are indistinguishable.

### *D*-statistics

We implemented *D*-statistics with *Dsuite* (Malinsky et al. 2021) across all combinations of the 16 macaque species described above. The topology required for *Dsuite* was consistent with the 2 most common tree topologies described in Figs. 1c and 2a and b. The *D*-statistics for all possible combinations with *P. anubis* as the outgroup (Out) and 3 different macaque species with a phylogenetic topology following (((M1, M2), M3), Out) were calculated using the Dtrios module. A total of 566 groupings were analyzed for each topology. *D*-statistics were calculated for each branch of alternative topologies using the *f*-branch module, and the statistical results were visualized using the dtools.py script provided with the *Dsuite* software.

### Detection of Hybridization

To obtain further support for ancient introgression as indicated by *D*-statistics, we applied HyDe (Blischak et al. 2018), which automates the detection of hybridization across large numbers of species, despite ILS, and can test a hypothesis at the population or individual level by estimating the amount of admixture (*γ*). The procedure is as follows: the population approach was used in the “run_hyde_mp.py” script to test hybridization events among all triplet combinations among the 4 macaque clades (*fascicularis*/*mulatta* clade, *arctoides/sinica* clade, *silenus/nigra* clade, and *sylvanus* clade) with *P. anubis* as the outgroup. The results were first filtered based on whether there was significant evidence of hybridization (*P* < 0.05). In addition, we estimated admixture levels using gamma (*γ*) statistics. Values of approximately 0.5 indicate a hybrid origin with a 50:50 contribution of both parental lineages, whereas extremely low or high values suggest the absence of hybridization events. We found that values of approximately 0.5 were significantly concentrated among the *silenus/nigra*, *fascicularis*/*mulatta*, and *arctoides/sinica* clades. Next, we used the script “run_hyde_mp.py” to test each individual within a putative hybrid population (*fascicularis*/*mulatta* clade) using specified triplets. We then performed a bootstrap resampling (500 replicates) of the individuals within the putative hybrid lineages for each specified triplet based on the “bootstrap_hyde.py” script.

### ML Inference for Reticulation with PhyloNet

We used the InferNetwork_MPL program in PhyloNet (Yu et al. 2014), which is based on the MPL algorithm, to analyze a set of every 3rd GF ML tree. In other words, 2,150 trees in a coalescent framework that accounts for ILS, while allowing for different numbers of reticulation events. To reduce the complexity and computational demand, we used only 1 individual per species. We set the number of reticulation events at 1 and 2 to conduct 2 independent analyses and each was run with 100 iterations to yielding 5 networks with the highest likelihood scores. We visualized the optimal networks in Dendroscope v3 (Huson and Scornavacca 2012).

### *F_d_* Value

To identify putatively introgressed regions in macaques, we followed the method described by Martin et al. (2015) and van der Valk et al. (2020). The method predicts that introgression between species in a specific genomic region should reduce the between-species divergence in this region compared with the rest of the genome (Martin et al. 2015; van der Valk et al. 2020). We computed the *F_d_* value, which is sensitive to the number of informative sites within windows, for each 10-kb window across the whole genome of each macaque.

### Introgression Analysis with Efficient Local Ancestry Inference

The 2-layer admixture model implemented in efficient local ancestry inference (Guan 2014) was used to infer introgressed segments in *fascicularis/mulatta* (the admixed population, -p 1) with *silenus/nigra* as source population 2 (-p 11) and *arctoides*/*sinica* as source population 1 (-p 10). We ran 30 separate iterations, setting the mixture generation values to 2.91 million, 3.2 million, and 3.45 million (based on the results of MCMCTree), and used the average of 3 independent runs to identify introgression events. Sites with a proportion of >1.5 from either source population were defined as introgression sites. We then calculated the sum of proportions >1.5 from each 10-kb window for each source population and defined the top 1% of the windows as putative introgression tracts.

### Analysis of Demographic History Using Genome-Wide Data

We performed a PSMC (v0.6.5-r67) analysis (Li and Durbin 2011) to reconstruct the demographic history of each macaque individual. To enhance mapping quality, we set parameter “-C” to 50 and created input files for PSMC using the SAMtools mpileup module (Li, Handsaker, et al. 2009; Li, Han, et al. 2009). Only autosomal SNVs were reserved to generate the diploid sequence for PSMC. PSMC was run with 25 iterations (-N), a maximum 2N0 coalescent time (-t) of 15, an initial theta/rho ratio (-r) of 5, and the 64 time intervals were parameterized as “4 + 25 ∗ 2 + 4 + 6.” PSMC plots were scaled with a mutation rate (*μ*) of 0.8 × 10^−8^ and a generation time (*g*) of 11 yr for all macaque species (Xue et al. 2016). Bootstrap analyses (100 bootstrap replications) were performed for each species.

### Divergence Time Calibration

Coding sequences (CDSs) for different representative outgroup species and 3 macaques (*M. mulatta*, *M. fascicularis*, *M. nemestrina*, *P. anubis*, *Homo sapiens*, *Pan troglodytes*, and *Pongo abelii*) were retrieved from Ensembl (release-98). The list of single-copy orthologous genes shared by these species was obtained by OrthoFinder (Emms and Kelly 2019). The macaques were mapped to the Panu_3.0 genome; thus, their CDSs have the same genomic coordinates. Therefore, the CDSs of all macaques were extracted after ortholog detection. CDSs of these single-copy genes were aligned with PRANK v.170427 (Löytynoja 2014) using the codon model and concatenated into a single supermatrix. Fourfold degenerate (4D) sites were extracted using MEGA v7 (Kumar et al. 2016) and concatenated to estimate the divergence times with MCMCTree in PAML v4.9 (Yang 2007). To calibrate the molecular clock, we set constraints on 4 nodes: Catarrhini 20 to 38 Ma, Hominidae 13 to 18 Ma, *Homo-Pan* 6 to 7 Ma, and *Papio*-*Macaca* 5.33 to 12.51 Ma (Perelman et al. 2011; Roos et al. 2019; de Vries and Beck. 2023). To check for convergence of the stationary distribution, the analysis was run in duplicate and the results of both runs were compared.

## Supplementary Material

msad229_Supplementary_Data

## Data Availability

Whole-genome resequencing data produced in this study were deposited at the NCBI Sequence Read Archive (SRA) under Bioproject PRJNA732172.

## References

[msad229-B1] Abbott R, Albach D, Ansell S, Arntzen JW, Baird SJE, Bierne N, Boughman J, Brelsford A, Buerkle CA, Buggs R, et al Hybridization and speciation. J Evol Biol. 2013:26(2):229–246. 10.1111/j.1420-9101.2012.02599.x. 23323997

[msad229-B2] Abbott RJ, Barton NH, Good JM. Genomics of hybridization and its evolutionary consequences. Mol Ecol. 2016:25(11):2325–2332. 10.1111/mec.13685. 27145128

[msad229-B3] Abi-Rached L, Jobin MJ, Kulkarni S, McWhinnie A, Dalva K, Gragert L, Babrzadeh F, Gharizadeh B, Luo M, Plummer FA, et al The shaping of modern human immune systems by multiregional admixture with archaic humans. Science 2011:334(6052):89–94. 10.1126/science.1209202. 21868630 PMC3677943

[msad229-B4] Árnason Ú, Lammers F, Kumar V, Nilsson MA, Janke A. Whole-genome sequencing of the blue whale and other rorquals finds signatures for introgressive gene flow. Sci Adv. 2018:4(4):eaap9873. 10.1126/sciadv.aap9873. PMC5884691 29632892

[msad229-B5] Avise JC, Robinson TJ. Hemiplasy: a new term in the lexicon of phylogenetics. Syst Biol. 2008:57(3):503–507. 10.1080/10635150802164587. 18570042

[msad229-B6] Blischak PD, Chifman J, Wolfe AD, Kubatko LS. Hyde: a Python package for genome-scale hybridization detection. Syst Biol. 2018:67(5):821–829. 10.1093/sysbio/syy023. 29562307 PMC6454532

[msad229-B7] Bouckaert RR . Densitree: making sense of sets of phylogenetic trees. Bioinformatics 2010:26(10):1372–1373. 10.1093/bioinformatics/btq110. 20228129

[msad229-B8] Castresana J . Selection of conserved blocks from multiple alignments for their use in phylogenetic analysis. Mol Biol Evol. 2000:17(4):540–552. 10.1093/oxfordjournals.molbev.a026334. 10742046

[msad229-B9] Chakraborty D, Ramakrishnan U, Panor J, Mishra C, Sinha A. Phylogenetic relationships and morphometric affinities of the Arunachal macaque *Macaca munzala*, a newly described primate from Arunachal Pradesh, northeastern India. Mol Phylogenet Evol. 2007:44(2):838–849. 10.1016/j.ympev.2007.04.007. 17548213

[msad229-B10] Ciani AC, Stanyon R, Scheffrahn W, Sampurno B. Evidence of gene flow between Sulawesi macaques. Am J Primatol. 1989:17(4):257–270. 10.1002/ajp.1350170402. 31964052

[msad229-B12] de Vries D, Beck R. Twenty-five well-justified fossil calibrations for primate divergences. Palaeontol Electron. 2023:26:a8. 10.26879/1249.

[msad229-B11] Delson E . Fossil macaques, phyletic relationships and a scenario of deployment. In: Lindburg DD, editor. The macaques. Studies in ecology, behavior, and evolution. New York: van Nostrand Rheinhold; 1980. p. 10–30.

[msad229-B13] Dierckxsens N, Mardulyn P, Smits G. NOVOPlasty: de novo assembly of organelle genomes from whole genome data. Nucleic Acids Res. 2017:45(4):e18. 10.1093/nar/gkw955. 28204566 PMC5389512

[msad229-B14] Durand EY, Patterson N, Reich D, Slatkin M. 2011. Testing for ancient admixture between closely related populations. Mol Biol Evol. 28(8):2239–2252. 10.1093/molbev/msr048. 21325092 PMC3144383

[msad229-B15] Edelman NB, Frandsen PB, Miyagi M, Clavijo B, Davey J, Dikow RB, García-Accinelli G, Van Belleghem SM, Patterson N, Neafsey DE, et al Genomic architecture and introgression shape a butterfly radiation. Science 2019:366(6465):594–599. 10.1126/science.aaw2090. 31672890 PMC7197882

[msad229-B16] Elton S, O'Regan HJ. Macaques at the margins: the biogeography and extinction of *Macaca sylvanus* in Europe. Quat Sci Rev. 2014:96:117–130. 10.1016/j.quascirev.2014.04.025.

[msad229-B17] Emms DM, Kelly S. 2019. Orthofinder: phylogenetic orthology inference for comparative genomics. Genome Biol. 20(1):238. 10.1186/s13059-019-1832-y. 31727128 PMC6857279

[msad229-B18] Evans BJ, Supriatna J, Andayani N, Setiadi MI, Cannatella DC, Melnick DJ. Monkeys and toads define areas of endemism on Sulawesi. Evolution 2003:57(6):1436–1443. 10.1554/02-443. 12894950

[msad229-B19] Evans BJ, Supriatna J, Melnick DJ. Hybridization and population genetics of two macaque species in Sulawesi, Indonesia. Evolution 2001:55(8):1686–1702. 10.1111/j.0014-3820.2001.tb00688.x. 11580028

[msad229-B20] Evans BJ, Tosi AJ, Zeng K, Dushoff J, Corvelo A, Melnick DJ. Speciation over the edge: gene flow among non-human primate species across a formidable biogeographic barrier. R Soc Open Sci. 2017:4(10):170351. 10.1098/rsos.170351. 29134059 PMC5666242

[msad229-B21] Fan P, Liu Y, Zhang Z, Zhao C, Li C, Liu W, Liu Z, Li M. Phylogenetic position of the white-cheeked macaque (*Macaca leucogenys*), a newly described primate from southeastern Tibet. Mol Phylogenet Evol. 2017:107:80–89. 10.1016/j.ympev.2016.10.012. 27769901

[msad229-B22] Fan Z, Zhao G, Li P, Osada N, Xing J, Yi Y, Du L, Silva P, Wang H, Sakate R, et al Whole-genome sequencing of Tibetan macaque (*Macaca thibetana*) provides new insight into the macaque evolutionary history. Mol Biol Evol. 2014:31(6):1475–1489. 10.1093/molbev/msu104. 24648498 PMC4032132

[msad229-B23] Fan Z, Zhou A, Osada N, Yu J, Jiang J, Li P, Du L, Niu L, Deng J, Xu H, et al Ancient hybridization and admixture in macaques (genus *Macaca*) inferred from whole genome sequences. Mol Phylogenet Evol. 2018:127:376–386. 10.1016/j.ympev.2018.03.038. 29614345

[msad229-B24] Feng S, Bai M, Rivas-González I, Li C, Liu S, Tong Y, Yang H, Chen G, Xie D, Sears KE, et al Incomplete lineage sorting and phenotypic evolution in marsupials. Cell 2022:185(10):1646–1660.e1618. 10.1016/j.cell.2022.03.034. 35447073 PMC9200472

[msad229-B25] Figueiró HV, Li G, Trindade FJ, Assis J, Pais F, Fernandes G, Santos SHD, Hughes GM, Komissarov A, Antunes A, et al Genome-wide signatures of complex introgression and adaptive evolution in the big cats. Sci Adv. 2017:3(7):e1700299. 10.1126/sciadv.1700299. 28776029 PMC5517113

[msad229-B26] Fontaine MC, Pease JB, Steele A, Waterhouse RM, Neafsey DE, Sharakhov IV, Jiang X, Hall AB, Catteruccia F, Kakani E, et al Extensive introgression in a malaria vector species complex revealed by phylogenomics. Science 2015:347(6217):6217. 10.1126/science.1258524. PMC4380269 25431491

[msad229-B27] Fontsere C, de Manuel M, Marques-Bonet T, Kuhlwilm M. Admixture in mammals and how to understand its functional implications: on the abundance of gene flow in mammalian Species, its impact on the genome, and roads into a functional understanding. Bioessays 2019:41(12):e1900123. 10.1002/bies.201900123. 31664727

[msad229-B28] Fooden J . Provisional classifications and key to living species of macaques (Primates: *Macaca*). Folia Primatol. 1976:25(2-3):225–236. 10.1159/000155715. 817993

[msad229-B29] Groves CP . Primate taxonomy. Washington DC: Smithsonian Institution Press; 2001.

[msad229-B30] Guan Y . Detecting structure of haplotypes and local ancestry. Genetics 2014:196(3):625–642. 10.1534/genetics.113.160697. 24388880 PMC3948796

[msad229-B31] Hamada Y, Urasopon N, Hadi I, Malaivijitnond S. Body size and proportions and pelage color of free-ranging *Macaca mulatta* from a zone of hybridization in northeastern Thailand. Int J Primatol. 2006:27(2):497–513. 10.1007/s10764-006-9033-4.

[msad229-B32] Hamada Y, Yamamoto A, Kunimatsu Y, Tojima S, Mouri T, Kawamoto Y. Variability of tail length in hybrids of the Japanese macaque (*Macaca fuscata*) and the Taiwanese macaque (*Macaca cyclopis*). Primates 2012:53(4):397–411. 10.1007/s10329-012-0317-3. 22875578

[msad229-B33] Heled J, Drummond AJ. Bayesian Inference of species trees from multilocus data. Mol Biol Evol. 2010:27(3):570–580. 10.1093/molbev/msp274. 19906793 PMC2822290

[msad229-B34] Hou W, Liu S, Jiang J, Fan Z, Fan P, Li J. The complete mitochondrial genome of white-cheeked macaque (*Macaca leucogenys*). Mitochondrial DNA B Resour. 2016:1(1):374–375. 10.1080/23802359.2016.1172039. 33473488 PMC7799460

[msad229-B35] Huson DH, Bryant D. Application of phylogenetic networks in evolutionary studies. Mol Biol Evol. 2006:23(2):254–267. 10.1093/molbev/msj030. 16221896

[msad229-B36] Huson DH, Scornavacca C. Dendroscope 3: an interactive tool for rooted phylogenetic trees and networks. Syst Biol. 2012:61(6):1061–1067. 10.1093/sysbio/sys062. 22780991

[msad229-B37] Ito T, Kanthaswamy S, Bunlungsup S, Oldt RF, Houghton P, Hamada Y, Malaivijitnond S. Secondary contact and genomic admixture between rhesus and long-tailed macaques in the Indochina Peninsula. J Evol Biol. 2020:33(9):1164–1179. 10.1111/jeb.13681. 33448526

[msad229-B38] Jablonski NG . The natural history of the doucs and snub-nosed monkeys. Singapore: World Scientific Press; 1998.

[msad229-B39] Kechin A, Boyarskikh U, Kel A, Filipenko M. Cutprimers: a new tool for accurate cutting of primers from reads of targeted next generation sequencing. J Comput Biol. 2017:24(11):1138–1143. 10.1089/cmb.2017.0096. 28715235

[msad229-B40] Kong S, Kubatko LS. Comparative performance of popular methods for hybrid detection using genomic data. Syst Biol. 2021:70(5):891–907. 10.1093/sysbio/syaa092. 33404632

[msad229-B41] Kumar S, Stecher G, Tamura K. MEGA7: molecular evolutionary genetics analysis version 7.0 for bigger datasets. Mol Biol Evol. 2016:33(7):1870–1874. 10.1093/molbev/msw054. 27004904 PMC8210823

[msad229-B42] Li H, Durbin R. Fast and accurate short read alignment with Burrows–Wheeler transform. Bioinformatics 2009:25(14):1754–1760. 10.1093/bioinformatics/btp324. 19451168 PMC2705234

[msad229-B43] Li H, Durbin R. Inference of human population history from individual whole-genome sequences. Nature 2011:475(7357):493–U484. 10.1038/nature10231. 21753753 PMC3154645

[msad229-B44] Li J, Han K, Xing J, Kim HS, Rogers J, Ryder OA, Disotell T, Yue B, Batzer MA. Phylogeny of the macaques (Cercopithecidae: *Macaca*) based on *Alu* elements. Gene 2009:448(2):242–249. 10.1016/j.gene.2009.05.013. 19497354 PMC2783879

[msad229-B45] Li H, Handsaker B, Wysoker A, Fennell T, Ruan J, Homer N, Marth G, Abecasis G, Durbin R. The sequence alignment/map format and SAMtools. Bioinformatics 2009:25(16):2078–2079. 10.1093/bioinformatics/btp352. 19505943 PMC2723002

[msad229-B46] Li C, Zhao C, Fan PF. White-cheeked macaque (*Macaca leucogenys*): a new macaque species from Medog, southeastern Tibet. Am J Primatol. 2015:77(7):753–766. 10.1002/ajp.22394. 25809642

[msad229-B47] Liu Z, Tan X, Orozco-terWengel P, Zhou X, Zhang L, Tian S, Yan Z, Xu H, Ren B, Zhang P, et al Population genomics of wild Chinese rhesus macaques reveals a dynamic demographic history and local adaptation, with implications for biomedical research. Gigascience 2018:7(9):giy106. 10.1093/gigascience/giy106. 30165519 PMC6143732

[msad229-B48] Löytynoja A . Phylogeny-aware alignment with PRANK. Methods Mol Biol. 2014:1079:155–170. 10.1007/978-1-62703-646-7_10. 24170401

[msad229-B49] Maddison WP, Knowles LL. Inferring phylogeny despite incomplete lineage sorting. Syst Biol. 2006:55(1):21–30. 10.1080/10635150500354928. 16507521

[msad229-B50] Malinsky M, Matschiner M, Svardal H. Dsuite—fast *D*-statistics and related admixture evidence from VCF files. Mol Ecol Resour. 2021:21(2):584–595. 10.1111/1755-0998.13265. 33012121 PMC7116594

[msad229-B51] Martin SH, Davey JW, Jiggins CD. Evaluating the use of ABBA-BABA statistics to locate introgressed loci. Mol Biol Evol. 2015:32(1):244–257. 10.1093/molbev/msu269. 25246699 PMC4271521

[msad229-B52] Martin SH, Van Belleghem SM. Exploring evolutionary relationships across the genome using topology weighting. Genetics 2017:206(1):429–438. 10.1534/genetics.116.194720. 28341652 PMC5419486

[msad229-B53] Matsudaira K, Hamada Y, Bunlungsup S, Ishida T, San AM, Malaivijitnond S. Whole mitochondrial genomic and Y-chromosomal phylogenies of Burmese long-tailed macaque (*Macaca fascicularis aurea*) suggest ancient hybridization between *fascicularis* and *sinica* species groups. J Hered. 2018:109(4):360–371. 10.1093/jhered/esx108. 29186474

[msad229-B54] McKenna A, Hanna M, Banks E, Sivachenko A, Cibulskis K, Kernytsky A, Garimella K, Altshuler D, Gabriel S, Daly M, et al The genome analysis toolkit: a MapReduce framework for analyzing next-generation DNA sequencing data. Genome Res. 2010:20(9):1297–1303. 10.1101/gr.107524.110. 20644199 PMC2928508

[msad229-B55] Meng G, Li Y, Yang C, Liu S. Mitoz: a toolkit for animal mitochondrial genome assembly, annotation and visualization. Nucleic Acids Res. 2019:47(11):e63. 10.1093/nar/gkz173. 30864657 PMC6582343

[msad229-B56] Morales JC, Melnick DJ. Phylogenetic relationships of the macaques (Cercopithecidae: Macaca), as revealed by high resolution restriction site mapping of mitochondrial ribosomal genes. J Hum Evol. 1998:34(1):1–23. 10.1006/jhev.1997.0171. 9467779

[msad229-B57] Patterson N, Price AL, Reich D. Population structure and eigenanalysis. PLoS Genet. 2006:2(12):e190. 10.1371/journal.pgen.0020190. 17194218 PMC1713260

[msad229-B58] Peng YZ, Pan RL, Jablonski NG. 1993. Classification and evolution of Asian colobines. Folia primatologica 60(1-2):106–117. 10.1159/000156680. 8335288

[msad229-B59] Perelman P, Johnson WE, Roos C, Seuánez HN, Horvath JE, Moreira MAM, Kessing B, Pontius J, Roelke M, Rumpler Y, et al A molecular phylogeny of living primates. PLoS Genet. 2011:7(3):e1001342. 10.1371/journal.pgen.1001342. 21436896 PMC3060065

[msad229-B60] Rogers J, Raveendran M, Harris RA, Mailund T, Leppälä K, Athanasiadis G, Schierup MH, Cheng J, Munch K, Walker JA, et al The comparative genomics and complex population history of *Papio* baboons. Sci Adv. 2019:5(1):eaau6947. 10.1126/sciadv.aau6947. PMC6401983 30854422

[msad229-B61] Roos C, Boonratana R, Supriatna J, Fellowes JR, Groves CP, Nash SD, Rylands AB, Mittermeier RA. An updated taxonomy and conservation status review of Asian primates. Asian Primates J. 2014:4:1–38. https://hub.hku.hk/handle/10722/198275.

[msad229-B62] Roos C, Kothe M, Alba DM, Delson E, Zinner D. The radiation of macaques out of Africa: evidence from mitogenome divergence times and the fossil record. J Hum Evol. 2019:133:114–132. 10.1016/j.jhevol.2019.05.017. 31358175

[msad229-B63] Roos C, Zinner D, Kubatko LS, Schwarz C, Yang M, Meyer D, Nash SD, Xing J, Batzer MA, Brameier M, et al Nuclear versus mitochondrial DNA: evidence for hybridization in colobine monkeys. BMC Evol Biol. 2011:11(1):77. 10.1186/1471-2148-11-77. 21435245 PMC3068967

[msad229-B64] Salzmann U, Williams M, Haywood AM, Johnson ALA, Kender S, Zalasiewicz J. Climate and environment of a Pliocene warm world. Palaeogeogr Palaeoclimatol Palaeoecol. 2011:309(1-2):1–8. 10.1016/j.palaeo.2011.05.044.

[msad229-B65] Sankararaman S, Mallick S, Dannemann M, Pruefer K, Kelso J, Pääebo S, Patterson N, Reich D. The genomic landscape of neanderthal ancestry in present-day humans. Nature. 2014:507(7492):354–357. 10.1038/nature12961. 24476815 PMC4072735

[msad229-B66] Sayyari E, Mirarab S. Fast coalescent-based computation of local branch support from quartet frequencies. Mol Biol Evol. 2016:33(7):1654–1668. 10.1093/molbev/msw079. 27189547 PMC4915361

[msad229-B67] Sinha A, Datta A, Madhusudan MD, Mishra C. Macaca munzala: a new species from western Arunachal Pradesh, northeastern India. Int J Primatol. 2005:26(4):977–989. 10.1007/s10764-005-5333-3.

[msad229-B68] Song Y, Jiang C, Li KH, Li J, Qiu H, Price M, Fan ZX, Li J. Genome-wide analysis reveals signatures of complex introgressive gene flow in macaques (genus *Macaca*). Zool Res. 2021:42(4):433–449. 10.24272/j.issn.2095-8137.2021.038. 34114757 PMC8317189

[msad229-B69] Sørensen EF, Harris RA, Zhang L, Raveendran M, Kuderna LFK, Walker JA, Storer JM, Kuhlwilm M, Fontsere C, Seshadri L, et al Genome-wide coancestry reveals details of ancient and recent male-driven reticulation in baboons. Science 2023:380(6648):eabn8153. 10.1126/science.abn8153. 37262153

[msad229-B70] Stamatakis A . RAxML version 8: a tool for phylogenetic analysis and post-analysis of large phylogenies. Bioinformatics 2014:30(9):1312–1313. 10.1093/bioinformatics/btu033. 24451623 PMC3998144

[msad229-B71] Steenwyk JL, Li Y, Zhou X, Shen XX, Rokas A. Incongruence in the phylogenomics era. Nat Rev Genet. 2023:1:17. 10.1038/s41576-023-00620-x. PMC11499941 37369847

[msad229-B72] Szöllősi GJ, Tannier E, Daubin V, Boussau B. The inference of gene trees with species trees. Syst Biol. 2015:64(1):e42–e62. 10.1093/sysbio/syu048. 25070970 PMC4265139

[msad229-B73] Thinh VN, Mootnick AR, Geissmann T, Li M, Ziegler T, Agil M, Moisson P, Nadler T, Walter L, Roos C. Mitochondrial evidence for multiple radiations in the evolutionary history of small apes. BMC Evol Biol. 2010:10(1):74. 10.1186/1471-2148-10-74. 20226039 PMC2841658

[msad229-B74] Tosi AJ, Disotell TR, Morales JC, Melnick DJ. Cercopithecine Y-chromosome data provide a test of competing morphological evolutionary hypotheses. Mol Phylogenet Evol. 2003:27(3):510–521. 10.1016/S1055-7903(03)00024-1. 12742755

[msad229-B75] Tosi AJ, Morales JC, Melnick DJ. Comparison of Y chromosome and mtDNA phylogenies leads to unique inferences of macaque evolutionary history. Mol Phylogenet Evol. 2000:17(2):133–144. 10.1006/mpev.2000.0834. 11083929

[msad229-B76] Tosi AJ, Morales JC, Melnick DJ. Paternal, maternal, and biparental molecular markers provide unique windows onto the evolutionary history of macaque monkeys. Evolution 2003:57(6):1419–1435. 10.1111/j.0014-3820.2003.tb00349.x. 12894949

[msad229-B77] Tung J, Barreiro LB. The contribution of admixture to primate evolution. Curr Opin Genet Dev. 2017:47:61–68. 10.1016/j.gde.2017.08.010. 28923540

[msad229-B78] Van der Auwera GA, Carneiro MO, Hartl C, Poplin R, Del Angel G, Levy-Moonshine A, Jordan T, Shakir K, Roazen D, Thibault J, et al From FastQ data to high confidence variant calls: the genome analysis toolkit best practices pipeline. Curr Protoc Bioinformatics. 2013:43(1):11.10.11–11.10.33. 10.1002/0471250953.bi1110s43. PMC4243306 25431634

[msad229-B79] Vanderpool D, Minh BQ, Lanfear R, Hughes D, Murali S, Harris RA, Raveendran M, Muzny DM, Hibbins MS, Williamson RJ, et al Primate phylogenomics uncovers multiple rapid radiations and ancient interspecific introgression. PLoS Biol. 2020:18(12):e3000954. 10.1371/journal.pbio.3000954. 33270638 PMC7738166

[msad229-B80] van der Valk T, Gonda CM, Silegowa H, Almanza S, Sifuentes-Romero I, Hart TB, Hart JA, Detwiler KM, Guschanski K. The genome of the endangered Dryas monkey provides new insights into the evolutionary history of the vervets. Mol Biol Evol. 2020:37(1):183–194. 10.1093/molbev/msz213. 31529046 PMC6984364

[msad229-B81] Wang K, Li M, Hakonarson H. 2010. ANNOVAR: functional annotation of genetic variants from high-throughput sequencing data. Nucleic Acids Res. 38(16):e164. 10.1093/nar/gkq603. 20601685 PMC2938201

[msad229-B82] Whelan NV, Kocot KM, Moroz TP, Mukherjee K, Williams P, Paulay G, Moroz LL, Halanych KM. Ctenophore relationships and their placement as the sister group to all other animals. Nat Ecol Evol. 2017:1(11):1737–1746. 10.1038/s41559-017-0331-3. 28993654 PMC5664179

[msad229-B83] Whitfield JB, Lockhart PJ. Deciphering ancient rapid radiations. Trends Ecol Evol. 2007:22(5):258–265. 10.1016/j.tree.2007.01.012. 17300853

[msad229-B84] Wu DD, Qi XG, Yu L, Li M, Liu ZJ, Yoder AD, Roos C, Hayakawa T, Rogers J, Marques-Bonet T, et al Initiation of the primate genome project. Zool Res. 2022:43(2):147–149. 10.24272/j.issn.2095-8137.2022.001. 35008130 PMC8920853

[msad229-B85] Xue C, Raveendran M, Harris RA, Fawcett GL, Liu X, White S, Dandouli M, Deiros DR, Below JE, Salerno W, et al The population genomics of rhesus macaques (*Macaca mulatta*) based on whole-genome sequences. Genome Res. 2016:26(12):1651–1662. 10.1101/gr.204255.116. 27934697 PMC5131817

[msad229-B86] Yamada KD, Tomii K, Katoh K. Application of the MAFFT sequence alignment program to large data-reexamination of the usefulness of chained guide trees. Bioinformatics 2016:32(21):3246–3325. 10.1093/bioinformatics/btw412. 27378296 PMC5079479

[msad229-B87] Yan G, Zhang G, Fang X, Zhang Y, Li C, Ling F, Cooper DN, Li Q, Li Y, van Gool AJ, et al Genome sequencing and comparison of two nonhuman primate animal models, the cynomolgus and Chinese rhesus macaques. Nat Biotechnol. 2011:29(11):1019–1023. 10.1038/nbt.1992. 22002653

[msad229-B88] Yang Z . PAML 4: phylogenetic analysis by maximum likelihood. Mol Biol Evol. 2007:24(8):1586–1591. 10.1093/molbev/msm088. 17483113

[msad229-B89] Yu Y, Dong J, Liu KJ, Nakhleh L. Maximum likelihood inference of reticulate evolutionary histories. Proc Natl Acad Sci U S A. 2014:111(46):16448–16453. 10.1073/pnas.1407950111. 25368173 PMC4246314

[msad229-B90] Zhang BL, Chen W, Wang Z, Pang W, Luo M, Wang S, Shao Y, He WQ, Deng Y, Zhou L, et al Comparative genomics reveals the hybrid origin of a macaque group. Sci Adv. 2023:9:22. 10.1126/sciadv.add3580 PMC10413639 37262187

[msad229-B91] Zheng Z, Wang X, Li M, Li Y, Yang Z, Wang X, Pan X, Gong M, Zhang Y, Guo Y, et al The origin of domestication genes in goats. Sci Adv. 2020:6(21):eaaz5216. 10.1126/sciadv.aaz5216. 32671210 PMC7314551

[msad229-B92] Zinner D, Arnold ML, Roos C. The strange blood: natural hybridization in primates. Evol Anthropol. 2011:20(3):96–103. 10.1002/evan.20301. 22034167

[msad229-B93] Zinner D, Fickenscher GH, Roos C. Family Cercopithecidae (old world monkeys). In: Mittermeier RA Rylands AB, Wilson DE, editors. Handbook of the mammals of the world. Volume 3, primates. Barcelona: Lynx Edicions; 2013. p. 550–627.

